# A conceptual review of mate choice: stochastic demography, within‐sex phenotypic plasticity, and individual flexibility

**DOI:** 10.1002/ece3.2197

**Published:** 2016-06-08

**Authors:** Malin Ah‐King, Patricia Adair Gowaty

**Affiliations:** ^1^ Centre for Gender Research Uppsala University Box 527 SE‐751 20 Uppsala Sweden; ^2^ Department of Ecology and Evolutionary Biology 621 Charles E. Young Dr. S. Los Angeles California 90095; ^3^ Department of Ethnology History of Religions and Gender Studies Stockholm University Universitetsvägen 10 E SE‐106 91 Stockholm Sweden; ^4^ Smithsonian Tropical Research Institute Box 0948, DPO AA 34002‐9998 Washington, D.C; ^5^ Institute of the Environment and Sustainability University of California Los Angeles California 90095

**Keywords:** Adaptive flexibility, choosy, genetic complementarity, indiscriminate, mate choice, OSR, parasite load, switch point theorem

## Abstract

Mate choice hypotheses usually focus on trait variation of chosen individuals. Recently, mate choice studies have increasingly attended to the environmental circumstances affecting variation in choosers' behavior and choosers' traits. We reviewed the literature on phenotypic plasticity in mate choice with the goal of exploring whether phenotypic plasticity can be interpreted as individual flexibility in the context of the switch point theorem, SPT (Gowaty and Hubbell 2009). We found >3000 studies; 198 were empirical studies of within‐sex phenotypic plasticity, and sixteen showed no evidence of mate choice plasticity. Most studies reported changes from choosy to indiscriminate behavior of subjects. Investigators attributed changes to one or more causes including operational sex ratio, adult sex ratio, potential reproductive rate, predation risk, disease risk, chooser's mating experience, chooser's age, chooser's condition, or chooser's resources. The studies together indicate that “choosiness” of potential mates is environmentally and socially labile, that is, induced – not fixed – in “the choosy sex” with results consistent with choosers' intrinsic characteristics or their ecological circumstances mattering more to mate choice than the traits of potential mates. We show that plasticity‐associated variables factor into the simpler SPT variables. We propose that it is time to complete the move from questions about within‐sex plasticity in the choosy sex to between‐ and within‐individual flexibility in reproductive decision‐making of both sexes simultaneously. Currently, unanswered empirical questions are about the force of alternative constraints and opportunities as inducers of individual flexibility in reproductive decision‐making, and the ecological, social, and developmental sources of similarities and differences between individuals. To make progress, we need studies (1) of simultaneous and symmetric attention to individual mate preferences and subsequent behavior in both sexes, (2) controlled for within‐individual variation in choice behavior as demography changes, and which (3) report effects on fitness from movement of individual's switch points.

## Introduction: From fixed sex‐typical strategies to within‐sex phenotypic plasticity to between‐individual flexibility

The literature on mate choice starting with Darwin (1871) is relatively large. Studies include elegant field experiments (e.g., Andersson 1992), clever laboratory experiments of crucial cues mediating mate choice (e.g.,Yamazaki et al. 1988; Dugatkin and Godin 1992b), labor intensive and inspired field observations (Forsgren et al. 2004; Jiggins et al. 2000), theory with intuitive predictions about the fitness payouts of mate choice (Hamilton and Zuk 1982), powerful phylogenetic studies of sexual signaling (Lynch et al. 2005), and well‐argued, interesting, and controversial alternative interpretations for observations (Breden 1988; Breden and Stoner 1988; Houde 1988; Stoner and Breden 1988).

Most empirical research on mate choice followed publication of William's (1966) cost of reproduction argument, Trivers' (1972) parental investment theory, and Parker's et al. (1972) anisogamy theory. These related ideas provided scenarios for the evolution of fixed, sex‐differentiated behavior due to posited ancient selection pressures acting on sex biases in gamete sizes and “parental investment.” Traditionally, therefore, investigators have assumed that mate choice is directional and fixed within a species, sex typical, and static within individuals over time and that choosers – usually females – chose mates on the basis of exaggerated, sexually selected traits – usually in males. As the current review shows, observations increasingly demonstrate that there is, in many species, considerable within‐sex phenotypic plasticity for choosy versus indiscriminate mating behavior (de Gaudemar 1998; Qvarnstrom et al. 2000; Forsgren et al. 2004; Plaistow et al. 2004; Lynch et al. 2005; Simcox et al. 2005; Lehmann 2007; Chaine and Lyon 2008; Heubel and Schlupp 2008; Ah‐King and Nylin 2010).

We wonder how much within‐sex phenotypic plasticity is actually among‐individual or even within‐individual flexibility. It is possible that individual flexibility is expressed independent of an individual's sex (Gowaty and Hubbell 2005, 2009), and it is certain that one cannot know whether this is the case without within‐species, within‐population symmetric tests on individuals of different sexes. It is also possible that the relatively commonly observed within‐sex phenotypic plasticity that we catalog here is really individual flexibility. We propose a conceptual transition to empirical studies of the inducers of individual flexibility (and the limits to flexibility) with renewed interest in the real‐time fitness effects of any observed flexibility.

## Background

Almost twenty‐five years ago, Hubbell and Johnson's (1987) discrete time mating theory (hereafter H&J's mating theory) opened the doors to tests of quantitative predictions of ecological and social constraints on individual flexibility in reproductive decisions. Their model provided analytical solutions to the expected mean and variance in lifetime mating success and, for the first time, an alternative to the parental investment hypotheses for choosy and indiscriminate behavior. Their results showed that the evolution of choosy and indiscriminate behavior of individuals depended on (1) probabilistic demography and (2) variation in the quality of mates. H&J's mating theory predicted adaptive phenotypic plasticity (see discussion in Gowaty and Hubbell 2005). Some authors used the concepts of H&J's mating theory to explore variation in mating behavior (Bjorklund 1990; McLain 1991, 1992; Michiels and Dhondt 1991; Travers and Sih 1991; Wickman 1992; Berglund 1993), and Crowley et al. (1991) produced a simulation model of individuals in a seasonal population based on the parameters associated with waiting to mate (being “choosy”), further inspiring research about environmental sources of variation in reproductive decisions. A later idea related to H&J's mating theory – but with important differences in assumptions – caught on and spread, inspiring many more empirical studies: Clutton‐Brock and Parker (1992) said that the potential reproductive rate (PRR) of the sexes determined the operational sex ratio (OSR) and, in turn, determined the opportunities for within‐sex competition over mates. Under the influence of PRR theory, investigators found compelling cases of “reversed sex roles” in choosy and indiscriminate behavior (Clutton‐Brock and Vincent 1991; Berglund 1994; Kvarnemo and Simmons 1998; Jirotkul 1999; Forsgren et al. 2004; Klug et al. 2008), so that females were called “the competitive sex” when males were rare and males “the competitive sex” when females were rare. Empirical discoveries stimulated additional continuous time models, which remained consistent with the cost of reproduction expectations and most often sought solutions to sex‐differentiated equilibrium conditions to predict mating rates as a function of costs of reproduction, population density, OSR, etc. The factors were hypothesized to affect the “direction of sexual selection” because each affected the relative rarity of one or the other sex. Today, there are dozens of papers reporting indiscriminate behavior in the *“*choosy sex” (Bjorklund 1990; Berglund and Rosenqvist 1993) and investigators and theorists have produced a large number of conceptual and theoretical explanations for observations of switches in which sex is “choosy.” Yet, few have concluded the obvious: *Within‐sex phenotypic plasticity is inconsistent with the predictions from the cost of reproduction arguments of fixed sex differences in reproductive decision‐making*.

A discrete time, analytical model, the switch point theorem (SPT) (Gowaty and Hubbell 2009) says that flexible individuals trade‐off time available for mating with fitness that would be conferred from mating with this or that potential mate, and it proved theoretically that individual flexibility in accepting potential mates on encounter (“indiscriminate” behavior) or rejecting potential mates and waiting for a better option (“choosy” behavior) is adaptive when demographic situations fluctuate or change. The SPT proved that adaptive flexibility increases an individual's expected lifetime reproductive success, irrespective of the sex of the individual, and thereby also proved that fixed choosy or indiscriminate mating behavior would be maladaptive and likely selected against. The parameters of the SPT are individual survival probability per unit time *s*, the probability of encountering potential mates per unit time during periods of receptivity *e*, the duration of any postmating time‐out or latency before reentering receptivity *o*, the number of potential mates *n*, and the distribution of fitness that would be conferred, the *w‐distribution*. The “switch point” is the point along an axis of ranked potential mates that indicates the fitness that would be conferred on a focal individual if they mated with a given potential mate. The ranks that a focal individual self‐referentially assigns (Box 1) to potential mates do not change: What does change is the demographic circumstances of the focal individual. For example, under variation in a focal individual's survival probability, his or her switch points between acceptable and unacceptable ranked potential mates may change: If the focal individual's survival probability increases, the switch point may move to better ranks so that the focal individual deems fewer potential mates acceptable and more unacceptable; if the focal individual's survival probability decreases, the switch point may move to potential mates with worse fitness ranks, so that the focal individual deems more potential mates acceptable, fewer unacceptable.

Box 1 **Glossary with definitions of inducing variables and terms indicating reproductive decisions and mating behavior**

***Accepting*** refers to the behavior of mating or accepting a mating solicitation; it may be associated with subtle motor patterns: simply staying still may be an acceptance signal (Markow 1987) or stereotypical postures or calls. Accepting a potential mating differs from appetitive behavior that may be associated with assessment of alternative potential mates.

***Assessment*** of alternative potential mates is a cognitive process and thus very difficult to operationalize or standardize. Ecologists and evolutionary biologists infer that individuals are assessing (something) by defined variation in appetitive or approach behavior. Neurobiologists may in the future evaluate assessment via imaging of neurological patterns.

***Consensus mate preference*** occurs when all or most individuals of one sex prefer the same opposite‐sex individual (which is in contrast to “individual” mate preference, defined below). For example, investigators of mallards inferred consensus mate preferences when female mallards displayed to dominant males on the wintering grounds (Cunningham and Russell 2000).

***Choosiness*** is defined as the effort an individual invests in mate assessment (Jennions and Petrie 1997), a definition without defined operational criteria.

***Choosy*** refers to the sensory ability of individuals to assess alternative potential mates or to motor patterns indicating rejection of some potential mates, but not others. In organisms in unrestricted field populations, investigators often assign the label “choosy” to subjects who reject some potential mates, but accept others. Like “choosiness,” “choosy” is a relatively loose term with many, often nonoverlapping meanings and is often difficult to operationalize, because it embeds and confounds cognitive and motor processes.

***Encountering a potential mate*** is a behavioral state of opposite‐sex individuals who are close enough for others to send or receive solicitation signals, rejection signals, or for individuals to otherwise sense characteristics of the potential mate. Empirical studies depend on operationalized definitions of “encountering” that may vary depending on the study species.

***Indiscriminate*** most often refers to individuals who accept copulations with alternative potential mates at random with respect to characteristics that investigators suspect are key traits choosers discriminate (songs, plumage, size, or other phenotypes). Thus, investigators' should perhaps label their subjects as “indiscriminate” relative to the particular tested traits in those being tested between.

***Individual flexibility*** refers to an extreme form of developmental variation, a type of plasticity induced by changing ecological and social circumstances of individuals in real time, not evolutionary time, and perhaps moment to moment. The term captures the idea that an individual may choose to do this or that or something else altogether, changing behavior moment to moment as circumstances change. It stresses the possibility of within‐individual changes, not just between‐individual changes. Many behavioral studies are about variation in individual behavior, for example, individual flexibility in foods taken, stored, and manner of retrieval.

***Individual mate preferences*** are those that are self‐referential so that preferences for potential mates are weighted or conditioned on the traits of the individual expressing “the preference.” Individual mate preferences could reflect “consensus mate preference” under some conditions. In practice, investigators of nonhuman animals infer “mate preferences” from subjects' behavioral variation, such as proximity to alternative potential mates, often in controlled situations such as “mate preference arenas”.

***Mate assessment*** is a cognitive evaluation based on individuals' abilities to sense differences between alternative potential mates, and in terms of Gowaty and Hubbell's (2009) switch point theorem (SPT), to rank alternative potential mates along chooser‐unique‐ranked axis of fitness that would be conferred by mating with any potential mate.

***Mate choice*** is a fuzzy term implying both cognitive and motor acts in which a focal individual accepts or rejects copulation with a potential mate. In practice, it is sometimes defined more narrowly as “any pattern of behavior, shown by members of one sex, that leads to their being more likely to mate with certain members of the opposite sex rather than others” (p. 4, Halliday, 1983). However, the later definition confounds mate preferences and/or mate assessments with other potential mediators of mating such as intrasexual interactions or sexual coercion.

***Preferences, including mate preferences,*** indicate cognitive states of an individual. Investigators characterize focal individual behavior — moving toward or orienting toward others, as indicating a preference for individuals or for individuals with different traits (e.g., plumage, calls). In other words, investigators infer cognitive states from behavioral correlates.

***Phenotypic plasticity*** is a term sometimes used to characterize moment‐to‐moment changes in phenotypes and thus overlaps in usage with individual flexibility. Here, we make a distinction between developmental conditions that induce usually fixed changes in phenotypes when individuals change sex in response to variation in the adult sex ratio. In contrast, individual flexibility is a term to indicate changes in behavioral phenotypes even moment to moment, as happens when individuals hide from predators. The color camouflage of octopus is an example of individual flexibility in moment‐to‐moment changes in phenotype.

***Rejecting*** refers to the behavior of individuals refusing to accept a copulation solicitation or a copulation attempt; it may be associated with failure to respond to copulation solicitation postures, or more active behavioral indicators, such as aggressive rejections or moving away from a soliciting opposite‐sex conspecific.

***Reproductive decisions*** refer to alternative motor acts of accepting a potential mate on encounter (which might appear “indiscriminate”) or waiting for a better mate (which might a appear as “choosy”). However, the SPT assumes that both decisions – either to mate on encounter or to wait for a better mate – are conditioned by an individual's prior assessment of the fitness that would be conferred by mating.

**Figure 1:**
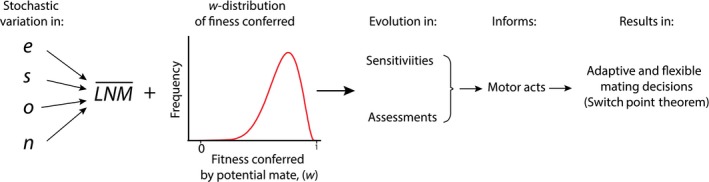
The evolution of adaptive, fitness enhancing, and flexible individuals (*the fourth column above*) able to switch their reproductive decisions based on their current demographic situations depends upon probabilistic (stochastic) variation in (*first column above*) a focal individual's encounter probability with potential mates, *e*, their survival probability *s,* the duration of any postmating time‐outs that the focal has experienced *o,* and the number of potential mates in the population *n*, which together predict an individual's expected mean lifetime number of mates under demographic stochasticity. *The second column above* indicates the SPT's explicit dependence upon the within‐population random distribution of fitness that would be conferred. *The third column above* indicates that the SPT assumes that selection occurred so that what evolved was (1) individual sensitivities to probabilities of encounter of potential mates *e,* probability of survival *s,* the duration of postmating time‐outs *o*, and the number of potential mates in the population *n* and the *w*‐*distribution* and in (2) abilities to assess the fitness that would be conferred by any potential mate. The SPT proved mathematically (*the fourth column above*) that individuals fixed in their reproductive behavior would be selected against relative to flexible individuals able to make real‐time mating decisions fit to their current ecological and social situations, as though decision‐makers are Bayesians able to update their priors to better fit their actions to the demographic and social circumstances they are in (Gowaty and Hubbell 2013).

The SPT changed the subject from sex‐specific behavior to individual‐specific behavior. It also changes the subject from the traits of the chosen sex to the social, ecological, and trait variation in the individuals doing the choosing. As we show here and as Gowaty and Hubbell (2005, 2009) argued, the scenario (Fig. 1) from the SPT for the evolution of flexible individuals potentially unifies and simplifies the large number of explanatory variables of empirically demonstrated within‐sex phenotypic plasticity in mate choice behavior (Table 1).

**Table 1:** The parameters of the SPT unify the phenomenological correlates of phenotypic plasticity in the behavior (motor acts) of accepting or rejecting potential mates usually called “being indiscriminate” or “being choosy”

Phenomenological predictors of switches	Inducing parameters of the SPT
Chooser's predation risk	Survival probability, *s*
Chooser's parasite load	
Chooser's condition	
Chooser's body size	
Chooser's age	
Chooser's resources	
OSR	Encounter probability, *e*
ASR	
Population density	
Territoriality	
Attractiveness of chooser	
Attractiveness of chooser's resources	
Audience effect	
Sperm competition risk	
Chooser's predation risk	
Chooser's parasite load	
Chooser's condition	
OSR	Number of potential mates, *n*
ASR	
Density of opposite‐sex conspecifics	
Chooser's predation risk	
Chooser's mating status (virgin or remating individual)	*o*
Chooser's predation risk	
Chooser's age	
Mate choice copying	*w‐distribution*
Audience effect	
Sperm competition risk	
Chooser's age	
Experience	

Studies of within‐sex mate choice plasticity usually focus on “choosy‐sex” behavior when the choosers vary in intrinsic characteristics such as age and experience, their condition, parasite load, and ecological and social circumstances, including the adult sex ratio (ASR), the operational sex ratio (OSR), density, and predator or parasite risk. Conclusions are thus necessarily about sexes and implied sex differences. But, many of these factors may induce within‐individual changes in behavior, and experiments of induced changes can provide evidence of individual flexibility in the ability to sense and respond in adaptive ways (Gowaty and Hubbell 2013) as individual's circumstances change. The factors tested in within‐sex mate choice plasticity studies seem relatively easy to measure, but there are a great many of such factors and some are obvious, complex proxies for the fundamental variables of the SPT (Fig. 1; Table 1).

Our goals with this review are to: (1) suggest how a few simple parameters unify and simplify a seemingly bewildering number of variables associated with within‐sex switches in choosy and indiscriminate behavior; (2) draw attention to environmentally induced behavior of individuals rather than sexes; and (3) propose how small methodological changes can evaluate how very simple variables may work to produce changes in behavior of individuals of either sex. We gathered papers testing within‐sex phenotypic plasticity in choosy and indiscriminate behavior (Table 2). We then categorized the variables in terms of their potential effects on the SPT's variables of probability of survival *s,* probability of encountering potential mates *e,* postmating time‐outs *o,* the number of potential mates in the population *n*, and the distribution of fitness that would be conferred *w‐distribution* (also in Table 2) and summarized the studies in various ways. Last, we discuss the implications of the reviewed studies taken together.

**Table 2:** Studies reporting within‐sex phenotypic switches from choosy to indiscriminate mating under variation in population density, OSR, ASR, chooser's condition, chooser's resources, predation risk, disease risk, and other factors sorted by the SPT's hypothesized inducers of individual flexibility: survival probability *s*, encounter probability *e,* duration of latency to remating *l,* the number of potential mates *n*, and the distribution of fitness that would be conferred under random mating, the *w‐distribution*

SPT	Predictor variable in study	Sex	Species	Citation	Dependent variable and response of the focal sex	Field or laboratory	Bias in PI	Both sexes as subjects
*s*	Age of chooser	♀	House crickets *Acheta domesticus*	Gray (1999)	Movement to male calls (attractive/unattractive). Young females chose the attractive call *(reject more)*, while older females did not express a significant preference *(accepted more)*.	Laboratory	♀	No
*s*	Age of chooser	♀	House crickets *Acheta domesticus*	Mautz and Sakaluk (2008)	Latency to mating. Older females had shorter latency to mating (time between male courtship and female mounting) *(accepted more)* than younger females *(rejected more)*.	Laboratory	♀	No
*s*	Age of chooser	♀	Tanzanian cockroaches *Nauphoeta cinerea*	Moore and Moore (2001)	Time of courtship until mating. Older females required shorter duration of courtship than younger females.	Laboratory	♀	No
*s*	Age of chooser	♀	Guppies *Poecilia reticulata*	Kodric‐Brown and Nicoletto (2001)	Movement toward video‐monitored male (plain/ornamented). Young females preferred more ornamented males *(rejected more),* and older did not differentiate with reference to male ornamentation *(accepted more)*.	Laboratory	♀	No
*e*	ASR	♀ ♂	Two‐spotted goby *Gobiusculus flavescents*	Forsgren et al. (2004)	“Over the short breeding season fierce male–male competition and intensive courtship behavior in males were replaced by female–female competition and actively courting females” (p. 551)	Field (obs)	♂	Yes
*e* and *s*	ASR and condition	♂	Three‐spined sticklebacks *Gasterosteus aculeatus*	Candolin and Salesto (2009)	Courtship intensity, number of leads to the nest. Without competition all males preferred large females, in male‐biased ASR high‐condition males kept their preference for large females, while low‐condition males did not discriminate (*accepted more*).	Laboratory	♀	No
*e*	Attractiveness of choosers' resources	♂	Beaugregory damselfish *Stegastes leucostictus*	Itzkowitz and Haley (1999)	Courtship to females that were experimentally placed in the field. Two types of artificial nest sites were distributed. Only males with the highest quality territories increased their courtship toward large females, males with lower quality territories showed similar low courtship intensity to large and small females *(accept more)*.	Field (experiment)	♀	No
*?*	Audience effect	♂	Atlantic molly, *Poecilia mexicana*	Plath et al. (2008a)	Proximity to either of two presented females (conspecific/heterospecific or large/small conspecific. Focal males spent less time near the initially preferred female and spent more time near the initially nonpreferred female when a conspecific audience male (that could not choose) was present.	Laboratory	♀	No
*?*	Audience effect	♂	Cave molly *Poecilia mexicana*	Plath et al. (2008b)	Proximity to either of two females (large/small). Focal males tended to divide their attentions more equally *(accept more)* between the two females when an audience male (that could not choose) was present.	Laboratory	♀	No
*?*	Audience effect	♂	Guppies *Poecilia reticulata*	Makowicz et al. (2010)	Courting either of two females (large/small). Males increased their courtship toward the large female when an audience male was present. The audience male showed no preference in relation to size (*accepted more*) after having watched the focal male interact with the large female, but after 24 h returned to prefer the large female.	Laboratory	♀	No
*?*	Audience effect	♂	Atlantic molly *Poecilia mexicana*	Bierbach et al. (2011a)	Proximity to female (large/small). Males ceased to show a preference when observed by other sexually active males (*accept more*).	Laboratory	♀	No
*s*	Body condition	♀	Zebra finches *Taeniopygia guttata*	Riebel et al. (2009)	Preference was determined by focal key picking for hearing song. Females in good condition (from small brood sizes) showed stronger preferences for call *(reject more)*.	Laboratory	♀	No
*s*	Body condition	♀	Three‐spined sticklebacks *Gasterosteus aculeatus*	Bakker and Mundwiler (1999)	Proximity to two monitors with virtual males courting (red/orange). Females with lower rearing condition preferred orange (less colorful) and high‐condition females preferred red males.	Laboratory	♀	No
*s*	Body condition	♀	Stalk‐eyed flies *Diasemopsis meigenii*	Cotton et al. (2006)	Acceptance or rejection of mating attempt. Females with larger eye span preferred large eye span males (*rejected more*), and females with small eye span showed no preference in relation to eye span size (*accepted more*).	Laboratory	♀	No
*s*	Body condition	♀	Dung beetle *Onthophagus sagittarius*	Watson and Simmons (2010)	Acceptance or rejection of randomly assigned mate. Large females were less likely to mate (*rejected more*).	Laboratory	♀/equal	No
*s*	Body condition	♂	Two‐spotted goby *Gobiusculus flavescens*	Amundsen and Forsgren (2003)	Time spent in proximity of female and no. courtship displays. Large males prefer colorful females, and small males show no preference in relation to coloring (*accepted more*).	Laboratory	♀	No
*s*	Body condition	Female	Swordtail fish, *Xiphophorus birchmanni*	Fisher and Rosenthal (2006)	Movement toward water‐born cues of well‐fed or food‐deprived males. Food‐deprived females show stronger preferences for well‐fed males.	Laboratory	♀	No
*s*	Body condition	♀	House sparrow, *Passer domesticus*	Griggio and Hoi (2010)	Association with either of two males (with enlarged vs. average throat patches). Females in poor condition show clear preference for average males compared to females in good condition who showed no clear preference.	Laboratory (aviary)	♀	No
*s*	Body condition	♀ e	Flies *Drosophila subobscura*	Immonen et al. (2009)	Occurrence of mating in assigned pairs of fed or food‐restricted individuals. Males provide a drop of regurgitated fluid before mating. Females in low condition (poorly fed) showed stronger preference for good condition (better fed) males.	Laboratory	♀ (‐MB?) nuptial gifts	No
*s*	Body condition	♀	Zebra finches *Taeniopygia guttata castanotis*	Burley and Foster (2006)	Proximity to males (red or green‐banded) in trial. Condition was reduced by trimming of feathers. Low‐condition females *accepted more* without reference to banding color.	Laboratory	♀	No
*s*	Body condition	♀	Wolf spiders *Schizocosa ocreata* and *S. rovneri*	Hebets et al. (2008)	Number of copulations with high‐ or low‐diet males (female presented with 2 males). High‐quality‐diet females *rejected more* than low‐quality‐diet females; low‐diet females accepted both high‐ and low‐diet males *(accepted more)*.	Laboratory	♀	NO
*s and e*	Body condition	♀	Pronghorn *Antilocapra americana*	Byers et al. (2006)	Mate search effort. After a dry summer, females were in low condition and a smaller proportion of females made an active mate sampling effort (*accept more*).	Field	♀	No
*s*	Body size	♂	Sockeye salmon *Oncorhynchus nerka*	Foote (1988)	Time spent in proximity of females and no. courtship displays in arenas. Males prefer females as big or bigger than themselves.	Laboratory	♀	No
*s*	Body size	♂	Poecilid fish *Brachyrhaphis rhabdophora*	Basolo (2004)	Proportion of time spent in proximity of potential mate (small/large). Large males preferred large females and small males small females.	Laboratory	♀	Yes^a^
*s*	Body size	♀	Swordtail fish *Xiphophorus multilineatus*	Morris et al. (2010)	Proximity to large courting male versus small sneaking male. Large size females have stronger preference for courting males *(rejected more)* compared to smaller females.	Laboratory	♀	No
*s*	Body size	♀	Swordtail fish *Xiphophorus cortezi, X. malinche*	Morris et al. (2006)	Proximity to either of 2 males (with symmetrical vs. unsymmetrical pigmentation). Larger (and older) females showed stronger preference for asymmetrical males.	Laboratory	♀	No
*s*	Body size	♀	African painted reed frog *Hyperolius marmoratus*	Jennions et al. (1995)	Movement toward speaker. Over all females preferred lower frequency. When presented with calls with small difference in frequency, larger females showed a bias toward low frequency calls and smaller females showed a bias toward a slightly higher frequency calls, possibly due to larger females body size making them more sensitive to variation in call frequencies.	Laboratory	♀	No
*w‐distribution*	Body size	♂	Sailfin molly *Poecilia latipinna*	Ptacek and Travis (1997)	Proportion of gonopodial nibbles and gonopodal thrusts toward large or small female. Larger males exhibited stronger preference for large females (rejected more)	Laboratory	♀	Yes^a^
*s, w‐distribution*	Body size	♂	Hermit crab *Pagurus middendorffii*	Wada et al. (2010)	1) Changing to a larger partner from the one currently guarded and 2) choice between two females presented simultaneously. Large males chose large females at all times (rejected more). Small males kept their smaller partner and balanced their preferences for large size with time to receptivity and thus more often chose a small partner close to spawning.	Laboratory	♀	No
*s, e, w‐distribution*	Body size	♀	Lizard *Lacerta vivipara*	Fitze et al. (2010)	Occurrence of copulation between focal female and males presented to her in succession. Larger females *rejected more* males before mating, and second mate was negatively correlated with size, that is*,* females chose a different size for their second mate.	Laboratory	♀	No
*s*	Body size, experience	♀	Swordtails *Xiphophorus nigrensis*	Wong et al. (2011)	Proximity to large versus small male. Female body size was positively correlated with preference for male size, *that is* larger females preferred larger males. Sexually experienced females showed stronger preference for large males compared to virgin females.	Laboratory	♀	No
e	Chooser attractiveness	♂	Three‐spined sticklebacks *Gasterosteus aculeatus*	Kraak and Bakker (1998)	The number of zigzags directed to and the time spent orienting to either of two females (large/small) presented simultaneously indicated male preference. Brighter but not dull males preferred larger females *(reject more)*.	Laboratory	♀	No
*w‐distribution*	Conspecific/heterospecific encounters?	♂	Sailfin molly *Poecilia latipinna*	Heubel and Schlupp (2008)	Proximity to conspecific versus heterospecific female. Males preference varied with season and they preferred conspecifics during breeding season *(rejected more)*. Otherwise, males mate also with asexuals *(Poecilia formosa) (accept more)*.	Laboratory	♀	No
*w‐distribution*	Context‐dependent mate choice	♀	Green swordtails *Xiphophorus helleri*	Royle et al. (2008)	Proximity to males in aquarium arena. Females were presented with males in three combinations: binary long sword/large body, three choices long sword/long sword/large body, or long sword/large body/large body. Females preferred the rare morph.	Laboratory	♀	No
*s*	Difference between spring and summer generation and age	♀	Real's wood white *Leptidea reali*	Friberg and Wiklund (2007)	Acceptance or rejection and time until acceptance. Spring and summer generations were manipulated to eclose at the same time. Females of different generations in this bivoltine butterfly differ in mating propensity. Spring females *reject more* males than summer females. Spring females also *accept more* as they grow older.	Laboratory	♀	No
*s*	Difference between spring and summer generation, time stress	♀ ♂	Green‐veined white *Pieris napi*	Larsdotter Mellström et al. (2010)	Acceptance or rejection and time to copulation. The two generations were manipulated to develop at the same time. Females of different generations in this bivoltine butterfly differ in mating propensity. Direct developing females (more time stressed) mate sooner (*accepted more*) than the diapause generation. (Males of the time stressed generation take longer to mate after eclosion as they are more immature at eclosion.)	Laboratory	♀	Yes
*e*	Encounter rate	♀	Swordtails *Xiphophorus birchmanni*	Willis et al. (2011)	Female association with conspecific/heterospecific male. Females were presented with conspecific and heterospecific males, varying time since last encounter with a conspecific male. Females preferred conspecifics when given a choice, but spent more time close to heterospecific after isolation from conspecifics (*accepted more*).	Laboratory	♀	No
*e*	Encounter rate	♂	Pipefish *Syngnathus typhle*	Berglund (1995)	Proximity to large/small female. Under high density of opposite sex, males chose large females. Under low density, males did not choose mate on the basis of size (*accepted more*).	Laboratory	♂	No
*e*	Encounter rate	♂	Goby *Chlamydogobius eremius*	Svensson et al. (2010)	Courtship intensity and proximity. Males that were presented with a small female immediately after a large female reduced their courtship intensity significantly. At lower encounter rate, males made no discrimination between large/small females	Laboratory	♀	No
*e*	Encounter rate, females deprived of males	♀	Mosquito fish *Gambusia holbrooki*	Bisazza et al. (2001)	All copulations occur by males forcibly copulating with females without any courtship. Proximity to males indicated preference. Females were more prone to stay close to males when male‐deprived. Postpartum females also spend more time closer to males compared to nondeprived females.	Laboratory	♀	No
*w‐distribution*	Experience	♀	Guppies *Poecilia reticulata*	Rosenqvist and Houde (1997)	Time spent near orange male. Females with experience of either only orange‐colored males or without color did not discriminate. But females with mixed experience preferred orange‐colored males (*rejected more*).	Laboratory	♀	No
*w‐distribution*	Experience	♀	Field crickets *Teleogryllus oceanicus*	Rebar et al. (2011)	Time to mounting and time retaining spermatophore. Females mated to an attractive male took longer to mate again and retained subsequent spermatophore for a shorter time.	Laboratory	♀	No
*w‐distribution*	Experience	♀	Lincoln's sparrows *Melospiza lincolnii*	Caro et al. (2010)	Behavioral response to playback. Females that heard low‐quality and high‐quality song in succession increased their activity in response to the latter.	Laboratory	♀	No
*e, n, w‐distribution*	Experience	♂	Red‐sided garter snakes *Thamnophis sirtalis parietalis*	Shine et al. (2006)	Time spent courting. Males that had been exposed to small females spent more time courting intermediate‐sized female than males that had met large females. Males from high‐density den preferred mating with large females (*rejected more*) while low‐density woodland males did not show preference with relation to size (*accepted more*).	Laboratory	♀	No
*w‐distribution*	Experience	♀	Wolf spider Schizocosa	Hebets and Vink (2007)	Experience enhances preference for brush‐legged males.	Laboratory	♀	NO
*w‐distribution*	Experience	♀	Butterfly *Bicyclus anynana*	Westerman et al. (2010)	Mating occurred with either of two morphological types: wild type or spotty. Inexperienced females preferred wild type. In contrast, females exposed to courtship by spotty males mated with spotty males.	Laboratory	♀	No
*w‐distribution*	Experience	♀	Variable field cricket *Gryllus lineaticeps*	Wagner et al. (2001)	Response to high/low chirp rate. Females were exposed to either high chirp rate or mixed chirp rates prior to testing preferences toward a sequence of low/high/low. The latter females reduced their response more to the last low chirp song.	Laboratory	♀	No
*w‐distribution*	Experience	♀	Fruit fly *Drosophila melanogaster*	Dukas (2005)	Percentage of females mated, courtship, and mating latency. Females with experience of courtship by small males were more likely to mate and had shorter courtship and mating latencies than females courted by large males.	Laboratory	♀	No
*w‐distribution*	Experience	♀	Amazon molly *Poecilia formosa*	Körner et al. (1999)	Proximity to videomonitored males of two heterospecific species (which both serve as sperm donors to this unisexual species), either the species a female was raised with or an unfamiliar species. Females tend to prefer males of the species they were raised with.	Laboratory	♀ (unisexual species)	No
*w‐distribution*	Experience	♀	Trinidad guppy *Poecilia reticulata*	Breden et al. (1995)	Proximity to male (colorful and noncolorful). Females raised with colorful males had stronger preference for colorful males than did females raised with noncolorful males or with no male.	Laboratory	♀	No
*e*	Experience	♂	Damselfly *Ischnurae legans*	van Gossum et al. (2001)	Male trying to clasp either of two female morphs. Males were habituated with either of the morphs. Males preferred the morph they had lastly been exposed to. The males were then exposed to the other morph in 2 days, and the preference was reversed.	Laboratory	♀	No
*w‐distribution*	Experience	♀	Wolf spider *Schizocosa rovneri*	Rutledge et al. (2010)	Female receptive response. Female juvenile experience of manipulated males (clear, black). Females avoided familiar male phenotypes and preferred those to which they had not been exposed.	Laboratory	♀	No
*s or e*	Experience	♀	Field crickets *Gryllus pennsylvanicus*	Judge (2010)	Occurrence of mating. Females with varying social experience (virgin without social experience/mated). Mated females preferred males with weaponry and older males. Inexperienced females *accepted more*.	Laboratory	♀	No
*s or e*	Experience	♀ and ♂	Wolf spider *Hogna helluo*	Wilder and Rypstra (2008)	Occurrence of mating depending on prior experience (encounters with opposite sex with or without mating). Females that had encountered but not mated were more prone to mate. Males that had encountered females but not mated were less prone to mate. Mated females were less prone to remate, and mated males were more prone to remate.	Laboratory	♀	Yes
*w‐distribution, s*	Experience, time to spawning	♀	Three‐spined sticklebacks *Gasterosteus aculeatus*	Bakker and Milinski (1991)	Duration of head‐up display with sequentially presented males (bright/dull). Females with increased experience of bright males decreased the duration of head‐ups for next male encountered (*rejected more*). When time to spawning comes close, females were less selective with respect to brightness (*accepted more*).	Laboratory	♀	No
*w‐distribution, s, e*	Experience, Time to egglaying	♀	Pied flycatchers *Ficedula hypoleuca*	Sirkia and Laaksonen (2009)	Pairing in the field with experimentally manipulating the order of settling in territories and manipulation of UV reflection. Females use multiple traits. UV coloration was used as a criterion early in the season but not late demonstrating time‐dependent female plasticity.	Field experiment	♀	No
*w‐distribution and s*	Experience and body size	♀	Field crickets *Teleogryllus oceanicus*	Bailey and Zuk (2009)	Female response to playback (movement to speaker). Females with experience of preferred song were less responsive (*rejected more*) than females with no acoustic experience. Larger individuals were more responsive (*accepted more*) and had higher likelihood of moving to speaker.	Laboratory	♀	No
*w‐distribution*	Experience of song	♀	Field crickets *Teleogryllus oceanicus*	Bailey and Zuk (2008)	Female response to playback. Individuals raised without experience of song were more responsive (*accepted more*) and had higher speed and likelihood of moving toward speaker. Experienced females were less responsive and more discriminating (*rejected more*).	Laboratory	♀	No
*w‐distribution*	Experience with opposite sex	♀	Bark beetles *Ips pini*	Roitberg et al. (1994)	Time spent with intermediate‐sized male after experience of either a group of small or a group of large males, and females preferred intermediate‐sized male if they had earlier experience of small males (*accepted more*).	Laboratory	♀	No
*w‐distribution*	Experience with opposite sex	♀	Wolf spider *Schizocosa*	Hebets (2003)	Pairing or rejection in mate choice trial. Females *accepted* familiar males *more* often and cannibalized unfamiliar males more often.	Laboratory	♀	No
*w‐distribution*	Experience with opposite sex	♀	Wolf spider *Schizocosa*	Hebets (2007)	Presence/absence of copulation. In age‐controlled subjects, experienced females preferred brush‐legged (ornamented) males and inexperienced did not show preference (*accepted more*).	Laboratory	♀?	No
*w‐distribution*	Experience with opposite sex	♂	Damselflies *Enallagma*	Fincke et al. (2007)	Reaction to individual glued to a stick (green female, blue female, or mature male). Males raised without experience of females had no bias toward either female morph, males with experience of one morph preferred that morph (*rejected more*). Study done in outdoor insectary	Laboratory	♀	No
*w‐distribution*	Experience with opposite sex	♂	Damselflies *Ischnura senegalensis*	Takahashi and Watanabe (2010)	Reaction to females glued to a stick (gyn‐ or andromorph). Males showed no bias toward either gyn‐ or andromorph in the morning, but were biased toward the most common morph in the afternoon (when experienced). Disappearance of bias during the night is suggested to be due to memory limitation.	Field	♀	No
*w‐distribution and l*	Experience with opposite sex and development	♀	Bushcricket *Steropleurus stali*	Bateman (2001)	Movement toward calling male. Females with mating experience decrease speed and movement toward calling males, compared to females without mating experience (*rejected more*).	Laboratory	♂	No
*w‐distribution and l*	Experience with opposite sex	♂	Field crickets *Gryllus bimaculatus*	Bateman and Fleming (2006)	Courtship effort. Males were presented with females (large and small) sequentially. Naive males treat large and small females equally. Males court more if presented with large female after small (*reject more*). When allowed to mate, males became more selective (*reject more*).	Laboratory	♀	No
*w‐distribution*	Experience of other's choice	♂	Pipefish *Syngnathus typhle*	Widemo (2006)	Display toward potential mates. Males but not females copied mate choice, that is*,* displayed toward females that were displayed to by other males.	Laboratory	♂	Yes^a^
*w‐distribution*	Experience of other's choice	♀	Ocellated wrasse *Symphodus ocellatus*	Alonzo (2008)	Mate choice copying, females more prone to mate if other females are around	Field	♂	No
*w‐distribution*	Experience of other's choice	♀	Guppies *Poecilia reticulata*	Briggs et al. (1996)	Time spent in preference zone of male. Female preferences were first determined (majority preferred bright males), after which they were given the opportunity to mate choice copy. Preferences were often reversed due to mate choice copying resulting in random preferences of male coloration.	Laboratory	♀	No
*w‐distribution*	Experience of other's choice	♀	Guppies *Poecilia reticulata*	Dugatkin (1992a)	Time spent in proximity of male close to model female or a sole male. A majority of females chose a male that had been accompanied by a female.	Laboratory	♀	No
*w‐distribution*	Experience of other's choice	♀	Sailfin molly *Poecilia latipinna*	Witte and Ueding (2003)	Time spent in proximity to male. Female preferences were first determined between two males. Preferences were reversed after showing a video in which another female escaped from the male that the female preferred. Thus, females copy rejection.	Laboratory	♀	No
*w‐distribution*	Experience of other's choice	♀ and ♂	Sailfin molly *Poecilia latipinna*	Witte and Ryan (2002)	Proximity to female and male versus one sole individual of opposite sex. Both males and females spent most time with the female and male, as in laboratory experiments, this experiment shows mate choice copying.	Field experiment	♀	Yes
*w‐distribution*	Experience of other's choice	♀	Three‐spined sticklebacks *Gasterosteus aculeatus*	Ridley and Rechten (1981)	Females accepting or rejecting potential mate. Females preferred to lay eggs in nest that already contained eggs.	Laboratory	♀	No
*w‐distribution*	Experience of other's choice	♀	Green anole lizards *Anolis carolinensis*	Stellar and White (2010)	Movement toward end chamber in which a large or small male had previously been presented. Females moved toward large male. When two females were tested simultaneously, one moved toward the large male and the other (subordinate) did not make a choice.	Laboratory	♀	No
*w‐distribution*	Experience of other's choice	♀	Three‐spined sticklebacks *Gasterosteus aculeatus*	Goldschmidt et al. (1993)	Mate choice copying, females were more prone to spawn with a male that already had eggs in the nest.	Field and Laboratory	♀	No
*w‐distribution*	Experience of other's choice	♀ and ♂	Three‐spined sticklbacks *Gasterosteus aculeatus*	Frommen et al. (2009)	Mate choice copying, females and males spent more time courting individuals that they had observed to be courted by others.	Laboratory	♀	Yes
*w‐distribution*	Experience of other's choice	♀	Guppies *Poecilia reticulata*	Godin et al. (2005)	Mate choice copying, females spend more time with males that have similar traits as males they have observed to be chosen by others.	Laboratory	♀	No
*w‐distribution*	Experience of other's choice	♀	Japanese quail *Coturnix japonica*	Galef and White (1998)	Mate choice copying. Female preference was determined by time spent in association with one of two males. Females that had watched their initially nonpreferred male mate with another female associated with him afterward.	Laboratory	♀	No
*w‐distribution*	Experience of other's choice	No	Japanese quail *Coturnix japonica*	White and Galef (1999)	Mate choice copying. Female preference was determined by time spent in association with one of two males. Females that had watched the male they initially did not prefer mate with another female, associated with him afterward.	Laboratory	♀	No
*w‐distribution*	Experience of other's choice	♀	Japanese quail *Coturnix japonica*	White and Galef (2000)	Mate choice copying, females spend more time with males that have similar traits as males they had observed to be chosen by others.	Laboratory	♀	No
*w‐distribution*	Experience of other's choice	♀	Japanese quail *Coturnix japonica*	Ophir and Galef (2004)	Mate choice copying, females increase their preference (time spent in proximity) for an initially nonpreferred male that they had seen copulating with another female.	Laboratory	♀	No
*w‐distribution*	Experience of other's choice	♀	Norwegian rats *Rattus norvegicus*	Galef et al. (2008)	Mate choice copying, females prefer to affiliate with a male that has recently engaged in sexual activity, even when they did not observe the male's previous mating.	Laboratory	♀	No
*w‐distribution*	Experience of other's choice	♀	Black grouse *Tetrao tetrix*	Höglund et al. (1995)	Mate choice copying, females are more likely to mate with a male that they have seen copulating with another female.	Field	♀	No
*w‐distribution*	Experience of other's choice	♀	Humpback limia *Limia nigrofasciata*	Munger et al. (2004)	Mate choice copying. Females are more likely to spend time in proximity with a male that they had seen in company with another female, then with their initially preferred male.	Laboratory	♀	No
*w‐distribution*	Experience of other's choice	♂	Sailfin molly *Poecilia latipinna*	Schlupp and Ryan (1997)	Mate choice copying of conspecifics and heterospecifics. Males copy mate choice. Initial preference (time spent in proximity) for conspecific female was reversed after another male was placed with the heterospecific female (gynogenetic associate, Amazon mollies *P. formosa*).	Laboratory	♀	No
*w‐distribution*	Experience of other's choice	♀	Zebra finch *Taeniopygia guttata*	Swaddle et al. (2005)	Mate choice copying, females significantly preferred individual males who had been paired with another female. Furthermore, a second experiment showed that females preferred novel males that were wearing the same leg band color as the apparently chosen males.	Laboratory	♀	No
*w‐distribution*	Experience of other's choice and hunger	♀	Guppies *Poecilia reticulata*	Dugatkin and Godin (1998)	Mate choice copying. Contrary to expectations, well‐fed but not starving females were more prone to spend time close to males that they had observed to be courted by others.	Laboratory	♀	No
*w‐distribution*	Experience of diet	♀	Predatory mite *Hypoaspis aculeifer*	Lesna and Sabella (1999)	Mating with males from either of two lines of males with genetic preferences for different prey. Females mated with dissimilar mates when fed on a mix of prey, that is*,* when hybrids are superior. When parental lines are superior, females mate assortatively within line.	Laboratory	♀	No
*w‐distribution*	Experience familiarity	♀	Decorated Crickets, *Gryllodes sigillatus*	Gershman and Sakaluk (2010)	Female retention time of sperm ampullae. (postcopulatory mate choice). Females preferred outbred and unfamiliar males, before familiar and inbred ones. Novel males of both inbred and outbred groups appeared equally attractive to females.	Laboratory	♀	No
*w‐distribution*	Experience familiarity	♀	Guppy *Poecilia reticulata*	Hughes et al. (1999)	Occurrence of mating. Females were more likely to mate with males having novel color patterns than with males having a color pattern with which they were familiar.	Laboratory	♀	No
*w‐distribution*	Experience familiarity	♀ and ♂	Guppy *Poecilia reticulata*	Zajitschek et al. (2006)	Occurrence of mating. Females preferred unfamiliar males. Males did not court unfamiliar females any more than familiar females and did not differentially allocate sperm.	Laboratory	♀	Yes
*w‐distribution*	Experience rearing	♀	Mallard *Anas platyrhynchos*	Kruijt et al. (1982)	Solicitations and responses. Females preferred males from their own rearing strain (even if cross‐reared wild/white).	Laboratory	♀	No
*w‐distribution*	Experience rearing	♀	Green swordtail *Xiphophorus helleri*	Walling et al. (2008)	Proximity to either of two males (long/short sword). Females were reared with (1) only long‐sworded, (2) only short‐sworded, or (3) a mix of long‐ and short‐sworded males. Only females with experience restricted to short‐sworded males developed any consistent preference, namely for short‐sworded males.	Laboratory	♀	No
*w‐distribution*	Experience rearing: Information during development	♀	Zebra finches *Taeniopygia*	Campbell and Hauber (2009)	Behavioral response and proximity to speakers with songs of same and different species. Females with experience of only same species did not differentiate between songs. Females with experience of different songs preferred their own species song (*rejected more*).	Laboratory	♀	No
*w‐distribution*	Experience rearing: Information during development	♂	Fruit flies *Drosophila paulistorum*	Kim et al. (1996)	Mating occurring or not. Individuals raised in isolation are indiscriminate (*accept more*).	Laboratory	♀	No
*w‐distribution*	Experience rearing: Information during development	♀ and ♂	Moths *Helicoverpa armigera*	Li et al. (2005)	Mating occurrence with individual fed on same larval host plant or different. Individuals fed on cotton preferred mating with cotton fed individuals. Peanut fed moths did not show preference in relation to host plant experience.	Laboratory	♀?	Yes
*l, s, w‐distribution*	Experience rearing: Rearing environment, virgin/mated, size, and age	♀	Swordtail fish *Xiphophorus malinche*	Tudor and Morris (2009)	Proximity to either of two presented males. Virgins did not show preference for symmetry. Rearing environment (symmetric, asymmetric or without bar pattern) influenced preference for symmetry. Female size affected strength of preference for symmetry *(larger females reject more)*.	Laboratory	♀	No
*w‐distribution*	Experience: Heterospecific rearing environment	♀ and ♂	Blue tit *Parus caeruleus* and great tit *Parus major*	Slagsvold et al. (2002)	Pairing. Cross‐fostering of blue tits and great tits resulted in female blue tits pairing with male great tits. However, females also copulated with blue tit males as all resulting offspring were blue tit. Great tits became imprinted on blue tits and failed pairing with conspecifics.	Field	♀	Yes
*?*	Experience (Sperm competition risk)	♂	Trinidadian guppies *Poecilia reticulata*	Jeswiet et al. (2011)	Proximity to either of two presented females. Sperm competition risk. Males change preference after seeing initially preferred female mate or are in proximity to another male guppy.	Laboratory	♀	No
*?*	Experience (Sperm competition risk)	♂	Eastern mosquito fish, *Gambusia holbrooki*	Wong and McCarthy (2009)	Proximity to either of two presented females. Sperm competition risk. Males change initial preference after seeing initially preferred female mate or be in vicinity of another male.	Laboratory	♀	No
*?*	Experience (Sperm competition risk)	♂	Atlantic molly *Poecilia mexicana*	Ziege et al. (2009)	Proximity to either of two presented females. Sperm competition risk. The strength of individual male preferences declined (*accepted more*) when another male was present (that could not choose).	Laboratory	♀	No
*w‐distribution*	Familiarity of potential mates	♂	Longsnouted seahorse *Hippocampus guttulatus*	Naud et al. (2008)	Proximity to potential mate. Males preferred larger unfamiliar mates before smaller familiar ones. No difference when choosing between unfamiliar and familiar of the same size, nor of larger and smaller unfamiliar ones.	Field and Laboratory	♀‐♂?	Yes^a^
*w‐distribution*	Frequency of different morphs (varying in viability by habitat)	♀	Soldier beetle, *Chauliognathus pennsylvanicu*	McLain (2005)	Frequency of mating. Changing frequency of male morphs changed female preference into positive density‐dependent preference.	Laboratory	♀	No
*s*	Food availability	♀	Black field crickets *Teleogryllus commodus*	Hunt et al. (2005)	Sexual responsiveness and strength of preference function (which calls females preferred). Females on high‐quality diet were more sexually responsive and had stronger preferences (rejected more) than females on low‐quality diets.	Laboratory	♀	No
*s*	Food availability and quality of developmental habitat	♀	Wolf spider *Pirata piraticus*	Eraly et al. (2009)	Probability of copulation. Individuals collected from polluted and unpolluted area, condition decreased by food deprivation. Food‐stressed unpolluted females decreased probability of copulation. Females from polluted area mated size assortatively, more strongly so under food stress.	Laboratory	♀	Yes
*s and l*	Food availability	♂	Bushcricket *Requena verticalis*	Kvarnemo and Simmons (1998)	Time in precopula, acceptance or rejection. A higher proportion of males that were fed a low‐protein diet rejected females, than males that were fed on a high‐quality diet.	Laboratory	♂‐♀	No
*s*	Food: Diet quality	♀	Canary *Serinus canaria*	Lerch et al. (2011)	Female solicitation displays. Diet influences preferences, females held on high‐quality diet showed stronger preferences of rapid tempo song, low‐quality diet (*accepted more*), had higher speed and likelihood of moving toward speaker.	Laboratory	♀	No
*s*	Food: Feeding status	♂	Insect parasitoid *Trichogramma turkestanica*	Martel et al. (2008)	Time spent in proximity to possible mates (virgin/mated). All males irrespective of feeding regime spent more time with virgin females, but unfed males had a higher proportion of time with virgin mates (*reject more*).	Laboratory	♀	No
*s*	Food: Feeding status, condition	♀	Cabbage looper moths *Trichoplusia ni*	Landolt et al. (1996)	Attraction to male pheromone. Fed females were less likely to move toward male pheromone (*rejected more*), compared to females that were not fed.	Laboratory	♀	No
*s*	Food: Hunger and age	♀	Wolf spider *Schizocosa ocreata*	Moskalik and Uetz (2011)	Responsiveness (latency to orient and receptive display) to video playback of modified courting male stimuli (larger/smaller body size, larger/smaller leg tufts). Long‐term starved females accepted large males, indifferent to small males with tufts and aggressive to small males with small tufts. Females that were starved during a short term were generally more receptive to all male types when older (*accepted more*). Satiated females accepted large males with ornaments, but less receptive to small males or males with small ornaments (*rejected more*).	Laboratory	♀	No
*s*	Food stress	♀	Stalk‐eyed flies *Cyrtodiopsis dalmanni*	Hingle et al. (2001)	Proportion of copulations with large or small eye‐spanned males (in cage on alternate days) in females fed on high‐ or low‐quality diets. High‐condition females preferred large eye‐spanned males *(reject more)*, low‐condition showed no preference *(accept more)*.	Laboratory	♀	No
*s and e*	Food: Variation in food and exposure to males	♀	Fruit fly *Drosophila melanogaster*	Chapman and Partridge (1996)	Remating frequency. Increasing nutrition increased remating frequency (*accepted more*) and was higher with continuous exposure to males.	Laboratory	♀	No
*s and e*	Food: Diet quality and ASR	♂	Bark louse *Lepinotus patruelis*	Wearing‐Wilde (1996)	Rate of solicitation/rejection. Males on high‐quality diets were significantly *more* likely to *reject* females than males on low‐quality diets. Among low‐quality diet males, those with large abdomens were *more* likely to *reject* females. Males were *more* likely to *reject* females under a female‐biased than a male‐biased adult sex ratio.	Laboratory	♂	Yes^a^
*s and e*	Habitat quality and body condition	♀	Galapagos marine iguanas *Amblyrhynchus cristatus*	Vitousek (2009)	Females *rejected fewer* males under poor food conditions than under better food conditions; females visited fewer males when in poor condition.	Field	♀	Yes^a^
*e*	Increased cost of choice	♂	Pacific blue‐eye *Pseudomugil signifer*	Wong and Jennions (2003)	Proximity to either of two females, without and with current. Males were consistent in their preferred (larger) mate without current (*reject more*), but inconsistent when exposed to current, thus increasing cost of staying close to initially preferred female (*accept more)*.	Laboratory	♀	No
*e*	Increased mate sampling cost against water current	♂	Guppies *Poecilia reticulata*	Head et al. (2010)	Displays and proximity to large/small possible mate. Males displayed less and were less discriminating *(accepted more)* on female size when in the presence of a water current compared to individuals in a still current	Laboratory	♀	Yes^a^
*e*	Increased mate sampling cost, experience	♀	Three‐spined sticklebacks *Gasterosteus aculeatus*	Milinski and Bakker (1992)	Female showing head‐up display. Females were selective when presented with dull and bright males sequentially. Females reduced their selectivity (*accepted more*) when costs of moving between males increased (swimming against a current). Females were also more prone to show head‐up display to a dull male *(accept more)* if first presented to a dull male rather than a bright one.	Laboratory	♀	No
*s*	Increased distance to possible mate	♀	Fiddler crab *Uca mjoebergi*	Booksmythe et al. (2008)	Movement toward waiving claw model, females were presented with two claw models and preferred the leading waiver if both were placed at the same distance, but the follower if it was closer than the leading waiver *(accept more)*. Trade‐off between preference and distance.	Field experiment	♀	No
*e*	Increased intrasexual competition (OSR)	♂	Spider *Zygiella x‐notata*	Bel‐Venner et al. (2008)	Male guarding or pairing in the field. During weak competition (balanced OSR) size of guarded and unguarded females did not differ, that is males did not preferentially guard large females (*accept more*). In high competition (male‐biased OSR), large males paired with large females and small males with small females.	Field	♀	No
*e*	Interaction and competition	♀ and ♂	Fruit flies *Drosophila pseudoobscura*	Kim et al. (2005)	Proximity to potential male versus occurrence of mating when competition and interaction was allowed (between two potential mates and the chooser). Both females and males preferred larger partners in nontouch testing arenas, but in actual mating when competition and interaction were allowed larger individuals did not mate more frequently.	Laboratory	♀	Yes
*w‐distribution*	Kinship variation	♀ and ♂	House finches *Carpodacus mexicanus*	Oh and Badyaev (2006)	Pairing in the field. Males and females were more closely related early in the season and less closely related later (and so were the population over all). Early in the season there was variation in male ornamentation, which was less late in season. Extra‐pair mates were less related than social mates both early and late in season.	Field observation	♀	Yes
*s*	Life span	♀	Cricket *Meloimorpha japonica*	Kuriwada and Kasuya (2006)	Female responsiveness to playback song. Females with a shorter life span exhibited higher responsiveness to the male calling song *(accepted more)* than females with long life span.	Laboratory	♀	No
*s, e or w‐distribution*	Light/dark	♀	Wolf spider *Schizocosa floridana*	Rundus et al. (2011)	Occurrence of mating in trials with one male and one female. Females mated equally often with low‐ and high‐quality‐diet males in the light environment, although in the dark treatment more often chose high‐quality‐diet males (*rejected more*). In the dark, seismic displays seem to become more important.	Laboratory	♀	No
*w‐distribution*	Offspring viability	♀ and ♂	Fruit flies *Drosophila pseudoobscura*	Gowaty et al. (2002)	Advancing toward potential mate. Both females and males were equally likely to advance toward (accept) and move away (reject). Number of offspring emerging as adults correlated with mutual interest (Sum of % toward vial mate movements: female toward + male toward).	Laboratory	♀	Yes
*e and s*	OSR	♀	Two‐spotted goby *Gobiusculus flavescens*	Borg et al. (2006)	Proximity to males (large/small). Early in season females accepted large males and rejected small males, and later they accepted males at random with respect to size as availability of males declined (*accepted more*).	Laboratory	♀?	No
*e*	OSR	♀	Guppies *Poecilia reticulata*	Jirotkul (1999)	Qualitative response of females to male sigmoid displays and the number of copulations with males varying in orange color. In male‐biased OSR, female preference for orange coloration is stronger.	Laboratory	♀	No
*e or n*	OSR	♂	Pipefish *Syngnathus typhle*	Berglund (1994)	Time spent in proximity of female, delay until courtship and copulation. In female‐biased OSR, males preferred large females, but in male‐biased sex ratio, males mated at random with respect to female size.	Laboratory	♂	No
*e*	OSR	♂	Flagfish *Jordanella floridae*	Klug et al. (2008)	Males *rejected more* females at higher encounter rates. Male aggression toward females higher when OSR was female biased.	Laboratory	♂	No
*e*	OSR	♂	katydids	Shelly and Bailey (1992)	Probability of mate acceptance or rejection. Males with low encounter rate (experimental and field caught) were more likely to mate than males with high encounter rate. Males with high encounter rate *rejected* lighter females *more*.	Laboratory	♂‐♀	No
*e*	OSR	♀ and ♂	Common goby *Pomatoschistus microps*	Borg et al. (2002)	Rejection or acceptance of females entering nest. Experimentally increased number of nests. During nest shortage, males *rejected more* often. Females courted more during nest shortage (*accept more)*.	Field experiment	♀	Yes
*e or n*	OSR	♀	Field cricket *Gryllus pennsylvanicus*	Souroukis and Murray (1995)	Proportion of courtships leading to mating. Females increased their acceptance rates as the sex ratio became female biased.	Field cage exp.	♀	N
*e or n*	OSR	♀ and ♂	Firefly *Photinus ignitus*	Cratsley and Lewis (2005)	Occurrence of mating. Females increased response as density of courting males decreased (*accepted more*). Male rejection of females increased as the season progressed and as male bias and female size decreased.	Field	♀	Yes
*e* or *s*	OSR variation induced by *Wolbachia* male killing	♀	*Acraea rncedon*	Jiggins et al. (2000)	Female swarms are analogous to male swarming butterflies. By analogy, males swarm to attract females; when the OSR is female biased, females swarm to attract males.	Field	FM	Yes
*e and s*	OSR, time to breeding	♂	Western grebe *Aechmophorus occidentalis*	Nuechterlein and Buitron (1998)	Response to heterospecific female call. Late‐courting males (male‐biased OSR) were more likely to court heterospecific females (*accept more*) than early‐courting (less male‐biased OSR) males (*reject more*).	Field playback experiment	♀	No
*e or n*	OSR, density	♀ and ♂	Striped ground cricket *Allonemobius socius*	Sadowski et al. (2002)	Occurrence of spermatophore transfer in different social settings: changing OSR/density of opposite‐sex individuals. Spermatophore transfer was more likely with more individuals than a pair. Females changed preferences (*accepted more*) with higher density. Male courtship was more intense when additional male or female was present.	Laboratory	♀	Yes
*s*	Oxygen stress	♀	Common goby *Pomatoschistus microps*	Reynolds and Jones (1999)	Spawning with either of two males, with or without eggs in nest. Females prefer to mate with males that already have eggs in their nest. But under oxygen stress, female preferences are reversed.	Laboratory	♀	No
*s*	Parasite load	♀	Upland bullies *Gobiomorphus breviceps*	Poulin (1994)	Time to visit and number of visits to two simultaneously presented males (large/small). Heavily parasitized females made fewer inspections and choose males randomly with respect to body size. Lightly parasitized females were more likely to choose the larger male.	Laboratory	♀	No
*s*	Parasite load	♂	Broad‐nosed pipefish *Syngnathus typhle*	Mazzi (2004)	Time spent in proximity of female. Sham‐infected males were more likely to mate with sham‐infected females rather than parasitized females. Infected males mated randomly with respect to female infection status.	Laboratory	♂	Yes^a^
*s and e*	Parasite load	♀	Guppies *Poecilia reticulata*	Lopez (1999)	Gliding toward showy/less showy male. Females with experimentally induced higher parasite load were less selective *(accept more)* and less active.	Laboratory	♀	No
*s*	Parasite load	♀	Calopterygid damselfly *Calopteryx haemorrhoidalis*	Cordoba‐Aguilar et al. (2003)	Occurrence of mating. Parasitized females spent less time during courtship and inspected fewer males before mating (*accepted more*).	field	♀	No
*s*	Parasite load	♀ and ♂	Bushcricket *Requena verticalis*	Simmons (1994)	Rejection of mating attempts. Uninfected females were discriminating *(rejected more)*, females infected (by protozoan gut parasite) attempted to mate more often (*accepted more*). Infection status in males did not affect male or female rejection rate.	Laboratory	♂‐♀	Yes^a^
*s*	Parasite load	♀	Couch's spadefoot toads *Scaphiopus couchii*	Pfennig and Tinsley (2002)	Movement toward speakers (long/short call). Uninfected females prefer long calls indicating male condition. Parasitized females had no preference for call duration (*accepted more*).	Laboratory	♀	No
*s and e*	Parasite load	♀	Wild turkeys *Meleagris gallopavo*	Buchholz (2004)	Mate inspection before mating. Infected females inspected more males before mating *(rejected more)* and had different preferences for snood length than noninfected females.	Laboratory	♀	No
*e or n*	Population density	♀	Kestrels *Falco thmuncuhts*	Palokangas et al. (1992)	Pair‐bonding in the field. When population density was high, females mated with long‐tailed mates, during years with low population density pairing was random with respect to tail length.	Field	♀	No
*e or n*	Population density	♀	Speckled wood butterfly *Pararge aegeria*	Gotthard et al. (1999)	Time to mating from first interaction with males. Females from low‐density population mated sooner *(accepted more)* than those from the high‐density population *(rejected more)*.	Laboratory	♀	No
*e or n*	Population density	♀ and ♂	Ladybeetles *Coleomegilla maculata*	Berglund (1994)	Frequency of remating by individuals kept isolated or in mix‐sex groups. Both females and males kept isolated had higher propensity to mate, this effect was larger in males.	Laboratory	♀	Yes
*e or n*	Population density	♀ and ♂	Fiddler crab *Uca uruguayensis*	Ribeiro et al. (2010)	Occurrence of mating. Density affects mating and searching: when density is low, mating occurs on the surface, when high mating is more often underground. Females differed in searching strategies between high‐ and low‐density sites, wandering around in high density and staying at own burrow in low. Mating males were larger in high density.	Field	♀	Yes
*e or n*	Population density and time available for mating	♀	Bushcrickets *Xederra charactus*	Lehmann (2007)	Rejection by walking away from potential mate. In high‐density populations, females encountered more males and *rejected more*; in low‐density populations, females encountered fewer males and *accepted more*. Females in low‐density populations were less likely to reject males later in the night when the mating period came close to an end.	Field	♀‐♂	No
*s*	Predation risk	♀	Crickets *Gryllus integer*	Hedrick and Dill (1993)	Moving toward short‐bout sound with cover or long‐bout sound without cover. Females prefer long‐bout sound. Increased predation made females move toward the short‐bout sound with cover instead *(accept more)*.	Laboratory	♀	No
*s*	Predation risk	♀	Guppy, *Poecilia reticulata*	Gong and Gibson (1996)	Female proximity to males in an arena with or without predator also visible. In the absence of predator, females preferred brighter males; in the presence of predator, females either did not exhibit any interest or preferred the duller male	Laboratory	♂	NO
*s*	Predation risk	♀	Sand gobies *Pomatoschistus minutus*	Forsgren (1992)	Proximity to one of two males (large/small, colorful/dull) presented with or without predator exposure. Females spent more time with large or colorful males in the absence of predators *(rejected more)*, in the presence of predator they *accepted* smaller and less colorful males.	Laboratory	♀	No
*s*	Predation risk	♀ and ♂	Black goby *Gobius niger*	Magnhagen (1990)	Occurrence of nesting and spawning. Black gobies did not nest and spawn under predation risk, whereas in the absence of predators, half of the males built nests and spawned.	Laboratory	♀	Yes
*s*	Predation risk	♀	Guppies *Poecilia reticulata*	Godin and Briggs (1996)	Proximity to one of two males (colorful/dull) presented with or without predator exposure. Females from high‐predation river reduced level of sexual activity and preference for a particular male during predation risk *(accept more)*. Females from low‐predation site did not change behavior when exposed to predator.	Laboratory	♀	No
*s*	Predation risk	♀	Tungara frogs *Physalaemus pustulosus*	Rand et al. (1997)	Female proximity to speakers with simple or complex call was recorded in dim light (higher predation risk) or darkness (lower predation risk). Females moved toward speakers more in the dark *(accepted more)* and more often toward a simple call close by than a more complex preferred call further away *(accepted more)*.	Laboratory	♀	No
*s*	Predation risk	♀	Tungara frogs *Physalaemus pustulosus*	Bonachea and Ryan (2011)	Female movement toward speakers with simple/complex call, combined with sound of predatory frog with the complex call and variation in light levels and perceived distance of call. Females were less prone to move toward complex preferred call combined with predator call *(accept more),* less likely to move long distances with predation risk, and made faster decisions with higher light levels (higher predation risk).	Laboratory and field	♀	No
*s*	Predation risk	♀	Fiddler crab *Uca mjoebergi*	Booksmythe et al. (2008)	Number of males rejected (bypassed) before visiting burrow were lower during increased predation risk (bird model) *(accept more)*.	field experiment	♀	No
*s*	Predation risk	♀	Fiddler crab *Uca beebei*	Kim et al. (2009)	Females visiting male burrows. Females preferentially visit males with large sand pillars that provide protection from predation, to male burrows without pillars. Under elevated predation risk females visited an even higher proportion of pillar builders (*reject more*).	field experiment	♀	No
*s*	Predation risk	♀	Fiddler crab *Uca terpsichores*	Kim et al. (2007)	Females visiting male burrows. Females preferentially visit males with large sand pillars that provide protection from predation. Under elevated predation risk, females visited an even higher proportion of pillar builders *(reject more)*.	field experiment	♀	No
*s and e*	Predation risk	♀ and ♂	Amphipods *Gammarus duebeni*	Dunn et al. (2008)	Likelihood of pair formation decreased and time to pair formation shortened with predation risk. Percentage of male antennation of females leading to pair formation increased and females resisted less with increased predation risk *(accept more)*. Males tended to mate more often with larger females at no predation risk *(reject more)*. Predation risk implies reduced activity, which leads to reduced encounter rate.	Laboratory	♀	Yes
*s and e*	Predation risk	♂	Broad‐nosed pipefish *Syngnathus typhle*	Berglund (1993)	Time spent dancing in front of female and number of copulations with large or small female was recorded in the presence/absence of predator. Males preferred large females in the absence of predator *(reject more)*, and during predation risk, they were less active and did not differentiate females with relation to body size *(accept more)*.	Laboratory	♂	No
*s*	Predation risk	♀ and ♂	Broad‐nosed pipefish *Syngnathus typhle*	Fuller and Berglund (1996)	Increasing predation risk reduces conspicuous mating behavior: time until first courting and number of matings decreased with higher predation risk and copulation occurred after a shorter time of courtship.	Laboratory	♂	Yes
*s*	Predation risk	♀	Field crickets *Gryllus rubens*	Velez and Brockmann (2006)	Motion toward speakers with male calls. Females of spring generation (with less predation risk by flies) readily move toward calling males *(accept more)*. Fall generation (under heavier predation risk) is less prone to approach callers *(reject more)*.	Laboratory	♀	No
*s*	Predation risk	♀	Green swordtails *Xiphophorus helleri*	Johnson and Basolo (2003)	Proximity to video with courting male indicated female preference for long‐sworded males on one side and digitally shortened on the other. Female preference for long‐sworded males changed after having seen video of long‐tailed male being eaten by predator indicating that females preferences for males with longer swords are modulated in the presence of a predator	Laboratory	♀	No
*s*	Predation risk	♀	Crickets *Acheta domesticus*	Csada and Neudorf (1995)	Moving toward synthesized optimal song without cover or suboptimal (increased period length) song without cover. Increased predation made females move toward less preferred song with cover instead *(accept more)*.	Laboratory	♀	No
*w‐distribution, s*	Predation risk and familiarity of females	♂	Panamanian bishop (poecilid fish) *Brachyrhaphis episcopi*	Simcox et al. (2005)	Mating attempts with familiar/unfamiliar females. In laboratory: males attempted to mate more with unfamiliar females, but did not distinguish between females when given only visual access. In field, males in populations with predation preferred unfamiliar females only when light levels were dim and not bright (at lower predation risk), males from low predation risk populations preferred unfamiliar females only during brighter but not dim conditions.	Laboratory and field	♀	No
*s*	Predation riskand diet	♀ and ♂	Water striders *Aquarius remigis*	Sih and Krupa (1992)	During predation risk, male harassment of females was lower and female resistance was more successful and therefore higher. Predation risk and food deprivation were experimentally manipulated.	Laboratory	♀	Yes
*s*	Predation risk and experience	♀	Atlantic mollies *Poecilia mexicana*	Bierbach et al. (2011b)	Proximity to small or large male. Inexperienced females preferred large males, but in the presence of a predator females changed to preferring small males. Wild‐caught females did not change their large male preference in the presence of predator.	Laboratory	♀	No
*s and e*	Predation risk and encounter rate	♀	Swordtails *Xiphophorus birchmanni*	Willis et al. (2010)	Female association with conspecific/heterospecific male. Females were presented with conspecific and heterospecific males, varying distance to shelter (predation risk) and varying time since last encounter with a conspecific male. Females preferred conspecifics when shelter was placed at equal distance to both males, but preferred heterospecific when shelter was closer to it (*accepted more*). Females also spent more time close to heterospecific after isolation from conspecifics (*accepted more*).	Laboratory	♀	No
*s, e, l or w‐distribution*	Rearing temperature/developmental form	♀ and ♂	Butterfly *Bicyclus anynana*	Prudic (2011)	Courtship rate, both sexes, and response to courtship (mating). Females developed during the dry season showed no preference for males with or without eyespot, females from the wet season preferred males with eyespot. In contrast, males developed during the dry season preferred females with eyespot and males from the wet season showed no preference. Larval rearing temperature cause shift in courtship and ornamental preference.	Laboratory	♀	Yes
*s and l*	Relative importance of male investment	♀ and ♂	Bushcricket *Requena verticalis*	Schatral (1993)	Rejection of mating attempts. Females *accepted more* when kept on a low‐protein diet due to greater need for male investment in nutritious spermatophore. Males *rejected more* when on a low‐protein diet, due to increased time to produce spermatophore and because females made more mating attempts.	Laboratory	♂‐♀	Yes
*e, l*	Relative importance of male parental investment	♀ and ♂	Katydids	Gwynne and Simmons (1990)	Frequency of rejection of mates (moving away from partner before spermatophore transfer). Increased pollen resources decreased female fighting over males and number of matings. Females *rejected more* potential mates when food was abundant. Increased pollen resources decreased male spermatophore production time, but also female need for nutritious spermatophores. Males *rejected more* when food was scarce.	Laboratory	♂‐♀	Yes
*w‐distribution*	Relatedness	♀ and ♂	House finches *Carpodacus mexicanus*	Lindstedt et al. (2007)	Occurrence of extra‐pair young. Social pairs were more related than extra‐pair pairs, for example individuals seek dissimilar mates for EPC. Both males and females solicited extra‐pair copulations in the absence of their social mate.	Field	♀	Yes
*s*	Reproductive stage	♀	Walnut‐infesting tephritid fly *Rhagoletis juglandis*	Carsten and Papaj (2005)	Occurrence of mating. Females with high egg loads were significantly more likely to copulate than low‐egg load females.	Laboratory	♀	No
*s*	Seasonal variation in benefit of burrow size	♀	Fiddler crab *Uca mjoebergi*	Milner et al. (2010)	Movement toward waiving large/small claw model. The strength of female preference of large clawed males shifts between summer and winter. Females overall prefer large clawed males, but the preference is weaker during winter, when larger burrows are not as important for optimal larvae growth.	Field exp	♀	No
*s, n, w‐distribution*	Time to breeding, age, available mates	♀	Wolf spiders *Schizocosa ocreata*	Uetz and Norton (2007)	Female sexual receptivity displays toward video‐recorded displaying male with enlarged/reduced/control ornament. Females showed preferences for enlarged ornaments early (*rejected more*) but no preference later (*accepted more*).	Laboratory	♀	No
*s*	Time to spawning	♀	Three‐spined sticklebacks *Gasterosteus aculeatus*	Luttbeg et al. (2001)	Probability of entering nest. Females were more prone to enter a nest when they had been held gravid for some time before making a choice. Females *accepted more* when they had been gravid a long time.	Laboratory	♀	No
*s*	Time to spawning	♀	Tungara frogs *Physalaemus pustulosus*	Lynch et al. (2005)	Response to call, acceptance of less attractive conspecific call and preference for whine‐chuck over whine. Females were most responsive (*accepted more*) when close to receptive stage.	Laboratory	♀	No
*s*	Time to spawning	♀	Tungara frogs *Physalaemus pustulosus*	Baugh and Ryan (2009)	Movement toward speaker, multiple trials. Females that were closer to egg laying were more consistent in their choice, *that is,* did not spend time on changing direction when more attractive calls appeared on the other side of arena.	Laboratory	♀	No
*w‐distribution*	Time of season	♀	Collared flycatchers *Ficedula albicollis*	Qvarnstrom et al. (2000)	Pairing in the field with males that had their forehead patch enlarged or unmanipulated. Females pairing early in the season did not mate preferentially with males with large forehead patches (*accepted more*), but females mating late did (*rejected more*). Also, female reproductive success is positively correlated with size of forehead patch only late in season.	field experiment	♀	No
*l*	Virgin/mated	♀	Field crickets *Gryllus pennsylvanicus*	Judge et al. (2010)	Time and occurrence of mating. Virgin females were quicker to mate (*accepted more*) than mated females.	Laboratory	♀	No
*l*	Virgin/mated	♀	Jumping spider *Evarcha culicivora*	Cross et al. (2007)	Proximity to large/small potential mate/occurrence of mating. Virgin females preferred larger males, and mated females preferred smaller and less dangerous males.	Laboratory	♀	Yes^a^
*l*	Virgin/mated and sequential matings	♀	Smooth newts *Triturus vulgaris vulgaris*	Gabor and Halliday (1997)	Occurrence of mating. Virgin females accepted mating with low‐/high‐ crested male (*accept more*). At second presentation, females only mated with higher‐crested male than in first mating (*reject more*).	Laboratory	♀	No

a Both sexes investigated in the study but changes in focal subject “choosiness” were found in only one sex as described in the table.

## Methods

To find studies on within‐sex phenotypic plasticity in choosy and indiscriminate mating behavior, we used Web of Science. We searched using phrases that we thought were common in the literature of changes in mating behavior including adult sex ratio or ASR, operational sex ratio or OSR, parasite load, predation risk, condition, age, and experience, each in combination with “mate choice or mate preferences,” as in “ASR and mate choice or mate preferences.” We also searched Web of Science for papers that cited early papers on mate choice flexibility: Losey et al. (1986), Hubbell and Johnson (1987), Kennedy et al. (1987), Houde (1987, 1988), Breden and Stoner (1987), Wade and Pruett‐Jones (1990), Shuster and Wade (1991), Clutton‐Brock and Parker (1992), Dugatkin (1992a), Pruett‐Jones (1992), and Hedrick and Dill (1993). The searches yielded over 3300 citations, of which 198 were empirical papers on changes in choosy versus indiscriminate behavior (Table 2). Box 1 contains a glossary with the meanings that we used for common terms. We categorized studies in Table 2 under probability of survival *s,* probability of encountering potential mates *e,* postmating time‐outs *o,* the number of potential mates in the population *n*, and the distribution of fitness that would be conferred *w‐distribution* (Gowaty and Hubbell 2009) depending on the information in each study. We coded studies of “audience effects” and “sperm competition risk” with question marks. We categorized some studies under multiple SPT parameters. In addition, 16 studies (Table 3) reported negative evidence of phenotypic plasticity.

**Table 3:** Negative studies that sought phenotypic plasticity, but found none

Authors	Species	Field/Laboratory	Result	Subjects
Aguilar et al. (2008)	Blue‐black grassquits *Volatinia jacarina*	Laboratory (captive wild‐caught birds)	Parasitized and nonparasitized females did not discriminate between males with and without parasites, although parasitism affected male display	♀
Plath et al. (2009)	Atlantic molly *Poecilia mexicana*	Laboratory	Female mating preferences are not changed by the presence of a conspecific or heterospecific female.	♀
Briggs et al. (1996)	Guppies, *Poecilia reticulata*	Laboratory	Predation risk did not influence frequency of mate choice copying in guppies. The study could not distinguish between mate choice copying or random mating.	♀
Eriksen et al. (2009)	Pied flycatchers, *Ficedula hypoleuca*	Field	Experience: Interspecific cross‐fostering affects song acquisition but not frequency of pairing success.	♀
Guevara‐Fiore et al. (2010)	Guppies, *Poecilia reticulata*	Laboratory	Inbreeding no effect. Inbreeding level does not induce female discrimination between sibs and unrelated males in guppies, that is inbreeding level did not influence female preference for unrelated males	♀
Hamilton and Poulin (1999)	Upland bully *Gobiomorphus breviceps*	Laboratory	Results illustrate both the variability among populations that prevents results obtained from one population from being generalized to the entire species, and the plasticity of sexually selected traits in relation to local conditions. Heavily parasitized females (field caught) take less time to first visit males than lightly parasitized or unparasitized females in one population, but reversed pattern in one other, and no effect in three populations.	♀
Head et al. (2010)	Guppies, *Poecilia reticulata*	Laboratory	No effect of water flow on female preferences. Females had no preference for male size or coloration when in a current or still water.	♀
Kelly et al. (2001)	Crustacia *Gammarus duebeni*	Laboratory	Females with sex ratio distorter do not differ in mating behavior from noninfected females. Males showed no preference for uninfected females, but mate‐guarded them for longer than infected females	♀♂
Lefebvre et al. (2005)	Amphipod *Paracalliope fluviatilis*	Field	Pair formation was random with respect to parasitism, but size assortative, and similar fecundity of infected and uninfected females	♀♂
Magellan and Magurran (2007)	Guppies *Poecilia reticulata*	Laboratory	Male sneak or display tactics in guppies are consistent over equal, female‐biased, and male‐biased sex ratios, that is males have individual mating behavior profiles	♀♂
Magnhagen (1990)	Sand gobies *Pomatoschistus minutus* black goby *Gobius niger*	Laboratory	Predation risk did not affect number of spawnings nor nests built by males in sand gobies; black gobies did not build nest or spawn in the presence of predators.	♀♂
Moore et al. (1996)	Rats	Laboratory	Experience of estrous odor – no effect on preference. After rearing by citral‐scented or unscented dams, adult male rats were given simultaneous choices of citral‐scented or unscented female partners. There was no evidence that mate choice had been affected by the early rearing experience.	♂
Pasteau et al. (2009)	Common Canaries *Serinus canaria*	Laboratory	Experience of song does not influence canary female preference for long phrases. Tested female preferences for “sexy phrases” of different durations. Two groups were used: (1) females raised in acoustic isolation and (2) females raised in “normal” acoustic conditions. No difference in preference, both groups prefer long phrases.	♀
Verzijden et al. (2009)	Lake Victoria Cichlids, *Pundamilia pundamilia* and *P. nyererei*	Laboratory	Cross‐fostering does not influence the mate preferences nor did territorial behavior of males	♂
Woodgate et al. (2010)	Zebra finches	Laboratory	Body condition in female zebra finches did not affect which males were chosen, but they were less active during choice trials and made fewer sampling visits to males	♀
Woodgate et al. (2011)	Zebra finches	Laboratory	Developmental stressors (nutritional stress) that impair song learning in males do not appear to affect female preferences for song complexity in the zebra finch.	♀♂

The justifications follow for placing common explanations (such as predation risk, mating status, OSR, condition, and age) into categories representing encounters with potential mates *e,* likelihood of survival *s,* duration of latency before reentering receptivity after mating *o,* the number of potential mates in the population *n*, and the likely fitness conferred from any mating or decision to accept a mating *w‐distribution. Predation risk* is an ecological correlate of changes from choosy to random mating (Breden and Stoner 1987). Predation risk logically may represent variation in probability of survival *s,* probability of encountering potential mates *e,* postmating time‐outs *o,* and the number of potential mates in the population *n* (Gowaty and Hubbell 2009) (Table 1). Predation risk very likely reduces individual instantaneous probability of *s*, but prudent prey may modify their behavior in the presence of predators, modifying their behavior to reduce their own conspicuousness, which is likely also to decrease their *e,* encounters with potential mates, as well as the local number of potential mates *n* that they or others may respond to. Experimental laboratory studies of predation risk almost always implicitly controlled for variation in probability of encounter of potential mates *e* and the number of potential mates in the population *n*, while remaining silent on variation in subjects' prior breeding experience, their ages, condition, and any reproductive success that might have accrued among individuals with different patterns of acceptances or rejections of potential mates. Thus, we categorized most studies of predation risk under probability of survival *s,* or probabilities of survival *s and* encounter *e* unless investigators provided other evidence that *e* or *n* varied (usually in studies of wild‐living subjects). *Age* (Kodric‐Brown and Nicoletto 2001) is intuitively important to reproductive decision‐making. But, age is a fuzzy proxy for an individual's probability of survival, *s*, and/or the effects of prior experience that can have effects on subjects' knowledge about the fitness that potential mates could confer, *w‐distribution*. In the studies of age effects in Table 2, investigators sometimes controlled for variation in experience. In models of individual flexibility in reproductive decision‐making, age is often correlated to variation in the duration of postmating time‐outs or latency, *o*, which in the absence of previous selection on choosy and indiscriminate mating will have no effect on virgins but will on nonvirgins. If virgins are always or often younger than nonvirgins, age may correlate with individual duration of time‐outs, *o*. Because virgins have never mated, the duration of time‐out is necessarily zero for virgins. We categorized studies that examined age effects on mate choice behavior under probability of survival, *s*. *Mating status* (Judge et al. 2010) effects on switches from choosy to random are still infrequently tested. However, in state‐dependent, discreet time models, such as H&J's mating theory (1987) or in the SPT (Gowaty and Hubbell 2009), the difference between virgins and mated individuals is captured with parameter *o,* the duration of postmating time‐outs. For virgins, the duration of postmating time‐outs always equals zero, effectively having nothing to do with the individual flexibility until after an individual's first mating, but for remating individuals, the duration of their postmating time‐outs *o* may be important. *If all else is equal*, that is, holding *e*,* s*,* n*, and the *w*‐*distribution* constant, the SPT and similar state‐dependent models predict that virgins mate on encounter more frequently than already‐mated individuals who are predicted more often to wait for a better option. Thus, we categorized studies investigating the effects of mating status, whether virgin or remating under duration of postmating time‐outs *o. OSR* (Berglund and Rosenqvist 1993) has been linked with within‐sex phenotypic plasticity in changes from accepting to rejecting potential mates. OSR may be a complex proxy for an individual's encounter probability with potential mates *e* as many investigators have argued, so we categorized studies of OSR with *e,* or under *e or n,* or *e and s* as *OSR* also contains information about number of potential mates and the instantaneous survival probability of decision‐makers. *Disease state, parasite load, condition, body size, and “attractiveness”* are usually linked not to choosers but to those individuals that choosers are assessing (Andersson 1994). More recently, focus has changed, so that investigators are asking whether variation in the “choosiness” of individuals of “the choosy sex” depends on the chooser's condition (Kodric‐Brown 1995), chooser's disease state or parasite load (Lopez 1999), or chooser's attractiveness (Itzkowitz and Haley 1999). Usually implicitly, investigators assume that condition, disease state, and parasite load affect within‐individual energy trade‐offs affecting the hypothesized costs of mate choice behavior. Intuitively, individual condition, disease state, body size, and parasite load may indicate variation in likelihood of survival, *s,* or the likelihood of encountering potential mates, *e*. We categorized condition, disease state, body size, and parasite load under *s, e,* or *s* and *e* depending on the information available in given papers. *Attractiveness of resources* (Itzkowitz and Haley 1999) sometimes affects chooser's encounters with potential mates; it might be a way that chooser's exploit the preexisting sensory biases of those they choose between, increasing their own encounters with potential mates by bringing potential mates to them in a passive way (West‐Eberhard 1984; Rodd et al. 2002). We categorized studies about the attractiveness of a chooser's territorial resources under probability of encounter with potential mates *e*. *Density* (McLain 1992) is a complex variable indicating the number of individuals in a given area; thus, it is possible that the salient inducers of change from rejecting to accepting potential mates are encounter probability *e* or the number of potential mates *n*. Whenever survival is negatively density dependent, parsing of density effects may require attention to the chooser's probability of survival *s* as well*. Mate choice copying* is a phenomenon that students of lekking species frequently speculated about even before Losey et al.'s (1986) model or the carefully controlled experiments of Dugatkin (1992a). We agree with Losey et al. that a key to the puzzle of mate choice copying is the fitness that would be conferred on a chooser versus copier females. Thus, we categorized mate‐copying studies under the fitness that would be conferred, *w‐distribution*.

## Results

Table 2 categorizes the 182 studies that reported evidence of phenotypic plasticity in “choosy” and “indiscriminate” behavior. Fifty‐four of the studies are on insects, eight on crustaceans, 13 on arachnids, 73 on fishes, seven on amphibians, four on lizards and snakes, 21 on birds, and two on mammals. Twenty‐eight of the studies were field studies, 150 were laboratory studies and four studies combined both laboratory and field components. One hundred and sixty‐nine were manipulative experimental studies and thirteen were observational studies describing how variation in ecological or social circumstances correlated with changes in focal subjects' “choosiness.” One hundred and twelve studies focused on females' mate choice behavior, 34 on males' mate choice behavior, and 36 on both sexes.

Within‐sex phenotypic plasticity (Table 2) occurred under changes in the OSR (12 studies), the ASR (three studies), population density (six studies), habitat/diet quality (15 studies), encounter rate (five studies), predation risk (20 studies), and changing cost of mate search (four studies). Also linked to within‐sex changes from choosy to indiscriminate mating behavior are: chooser's condition (21 studies, seven of which were on parasite load), chooser's body size (11 studies), chooser's resources (two studies), chooser's attractiveness (one study), chooser's experience (67 studies), mating status, that is, virgin/mated (four studies), and chooser's age (eight studies).

We provisionally categorized Table 2 studies under the SPT parameters: 84 under the focal chooser's probability of survival *s;* 55 under chooser's probability of encounter with potential mates *e*, 11 under the number of potential mates *n*, 10 under the duration of postmating time‐outs or latency *o,* and 76 by the *w*‐*distribution* (and we categorized some studies under multiple SPT parameters).

In addition to the Table 2 studies, we found 16 studies (Table 3) that looked for but did not find evidence of phenotypic plasticity.

## Discussion

From Table 2, one may conclude that within‐sex phenotypic plasticity in choosy versus indiscriminate behavior is (1) easily manipulated in laboratory studies and relatively easy to observe under naturally occurring conditions in the field. (2) Within‐sex phenotypic plasticity is common, widespread taxonomically occurring in insects, spiders, crabs, birds, amphibians, and fish. (3) Individuals of “the choosy sex” were phenotypically plastic in tested species and sometimes at random with respect to traits in the chosen sex usually suspected of evolving under sexual selection. Of the 198 studies reviewed here, only 16 (Table 3) reported no within‐sex phenotypic plasticity in reproductive decision‐making. Either within‐sex phenotypic plasticity is very common, at least wherever investigators look for it, or there is some remaining publication bias against studies that looked for but did not find within‐sex phenotypic plasticity of reproductive decisions. Almost all studies provisionally fit one or more of the SPT's simplifying parameters of survival probability *s,* probability of encountering potential mates *e,* the number of potential mates in the population *n,* the duration of postmating time‐outs or latencies to receptivity *o,* and the distribution of fitness that would be conferred *w‐distribution*.

### Chooser's traits and their social and ecological circumstances matter

Table 2 shows that in a variety of species and situations, variation in subjects' mating decisions depends as much or perhaps more on choosing subjects' intrinsic variation and their ecological and social situations as on the apparent variation in those being chosen between (which receives the most attention in studies of the evolution of traits, mostly in males). The review shows that choosers, both female and male, can and do modify their reproductive decisions under ecological and social contingencies that often have little or nothing to do with traits of chosen individuals. Most notable perhaps was that the ecological conditions or intrinsic variation of the subjects doing the choosing seemed often to matter more than characteristics of individuals among whom choosers' discriminated.

The SPT explicitly makes the point that the social and ecological situation (expressed as the focal individual's probability of survival *s*, probability of encounter with potential mates *e,* the duration of any postmating time‐outs *o,* the number of potential mates in the population *n,* and *w*‐*distribution*, Fig. 1) of the focal individual determines their reproductive decisions to accept or reject a given potential mate. The SPT not only changes the subject to individuals making reproductive decisions, but also takes attention off of the supposedly sexually selected traits of those being chosen. Instead of the usual attention to fancy male traits that supposedly exploit female choosers' sensory biases, the SPT refocuses on the fitness that would be conferred *on the chooser* under time costs rendered by variation in chooser's own likelihood of survival, their probability of encounters with potential mates, and the durations of any postmating time‐outs they have experienced. Paying attention to choosers' probability of survival brings attention to *chooser's* health status, *chooser's* parasite loads, *chooser's* current risk of being predated, and *chooser's* fancy traits that increase their probability of encounters with potential mates, which the studies here have also attended to but from different organizing perspectives than that of the SPT.

The parameters: the focal individual's probability of survival *s*, probability of encounters with potential mates *e*, the number of potential mates in the population *n*, the duration of any postmating time‐outs *o,* and the distribution of fitness that would be conferred, the *w‐distribution*, either separately or together (Table 1), may explain the results of a large number of the studies in Table 2. The SPT argues that a specific type of trade‐off underlies switches between acceptable and unacceptable mates, namely the trade‐off between an individual's real‐time fitness accrual and time available for mating. And, the SPT parameters are simpler than complex proxies such as OSR, “predator risk,” or “sperm competition risk.” For example, OSR may not adequately capture the inducing variable of individual behavior: Even if there are more sexually available females than males or males than females, the rarer sex may flexibly adjust reproductive decisions given their own probability of survival *s*, their probability of encounters with potential mates *e,* the number of potential mates in the population *n*, or the *w‐distribution*. Likewise, predator risk is complex, because the number of potential mates *n* may decline in the presence of a predator, an individual's immediate survival probability *s* surely decreases, and their encounters with potential mates *e* may decline. The SPT has shown that there is a hierarchy among inducing variables that have an order of magnitude or more effect on expected lifetime reproductive success. For example, individuals' survival probability, *s*, has a relatively huge effect compared to probabilities of encounter with potential mates on expected mean lifetime mating success and individuals' reproductive decisions: Theoretically, when an individual's probability of survival *s* declines, choosy individuals may accept potential mates that were previously unacceptable. Similarly, if an individual's encounter probability *e* with potential mates increases enough, they may reject potential mates that were previously acceptable. Sensitivity analyses (Gowaty and Hubbell 2009) of the SPT indicated that variation in survival probability theoretically has a significantly bigger effect than encounter probability on the expected mean lifetime mating success of an individual and thus on their switch points. In the case of the complex proxies reviewed herein, we argue that what may be inducing changes in individuals' reproductive decisions may be their probabilities of survival – *s*, probabilities of encounters with potential mates – *e,* the durations of their postmating time‐outs – *o,* variation in the number of potential mates – *n,* or the *w‐distribution*.

### A sea change from Darwin's observations of coy females and indiscriminate males

Widespread evidence of within‐sex phenotypic plasticity in choosy and indiscriminate behavior is quite different (Knight 2002) from Darwin's observation of coy females and indiscriminate males. In contrast to expectations from anisogamy and parental investment hypotheses, within‐sex phenotypic plasticity in reproductive decisions – to accept or reject potential mates – changes according to availability of potential mates, ecological conditions, social interactions, and the attractiveness and/or health status of the choosing individual or the resources available to the choosing individual. Yet, so far we can only safely conclude that subjects of the expected or usual “choosy sex” differ, which allows the conclusion of phenotypic plasticity among individuals in “the choosy sex”, but says nothing about individuals in “the indiscriminate sex.” How often is the “indiscriminate sex” phenotypically plastic? An empirical answer is unlikely as long as investigators confine their questions to individuals of “the choosy sex,” because observations of phenotypic plasticity in one sex are uninformative about the other sex.

Most if not all of the observations of and conclusions about within‐sex phenotypic plasticity are inconsistent with parental investment and anisogamy theories predicting fixed sex differences in mate choice behavior because of ancient selection pressures on gamete morphology or parental investment patterns.

We argue for consideration of an alternative possibility that within‐sex plasticity is actually individual flexibility, meaning that individuals rather than being “fixed” use the same rules of induced behavior, and alter their reproductive decisions regardless of their sexes, to fit current demographic and social circumstances, so that their behavior is individually flexible and adaptive in their current environments.

### Are there fitness consequences of switching between choosy and indiscriminate mating behavior?

The answer is impossible to tell from the studies in Table 2. The SPT (Gowaty and Hubbell 2009) like the earlier H&J mating model (1987) predicts the expected reproductive success for an individual switching from accepting to rejecting potential mates. Thus, it is worth mentioning that very few investigators have tackled empirically questions about fitness costs and benefits to individuals that switch from choosy to random mating, which is particularly interesting given that we were able to categorize 42% of the reviewed studies as provisionally being explained by variation in the fitness that would be conferred, that is the *w*‐*distribution*. We find most curious the absence of attention to the fitness payouts or relative fitness costs to benefits of switching in the remarkably sophisticated empirical studies of mate choice copying. Losey et al. (1986) said almost 30 years ago *“*…copying may be advantageous. Copying may also result in all the other females copying a poor choice. We can find no empirical or theoretical demonstration of the relative fitness of these two alternatives strategies in the literature (p 654)”. Losey et al. then provided a theoretical answer: if chooser females do choose fitter males and if choosers are more common than copiers, the fitness differences between chooser and copier females may be a wash. Since then, a game theoretic model has demonstrated that the adaptive significance of mate choice copying could reside in the ratio of costs and benefits to “active mate searching” (Pruett‐Jones 1992), which the SPT expresses as variation in probabilities of survival *s* and/or probability of encounters with potential mates *e* or *s* and *e*. And, the question remains: Are there fitness consequences of being a chooser versus a copier?

### Implications of variability in methods of laboratory and field studies

Methods and criteria for demonstrating variation in “the choosy sex” depend on the study species, whether the study was experimental or observational, field, or laboratory study (Table 2). By design, laboratory studies are relatively more similar in methods of evaluating individual subjects' decisions to accept or reject particular mates. Most laboratory studies of mate preferences depend on controlled testing conditions to exclude the simultaneous operation of within‐sex interactions and between‐sex coercion on the “preference” behavior of subjects (Kingett et al. 1981; Lambert et al. 1982); most use measures of approach (counts) or duration of time in proximity to assign relative preferences of a focal individual. In contrast, the operational definitions (Box 1) indicating “mate choice,” which investigators use under more complex social conditions where it is difficult or impossible to eliminate same‐sex interference or sexually coercive behaviors, are far more variable than the operational definitions used in laboratory studies. Thus, in practice, investigators use different definitions of “mate choice” and “mate preferences”. In Table 2, some studies used proximity to a potential mate or to alternative potential mates, moving toward potential mates, time near alternative potential mates, “courtship signals,” or mating with alternative potential mates, time from introduction to courtship or mating. Investigators usually attempt to adjust any behavioral indicator of a given reproductive decision to the behavior of the study organism, but these different definitions present a number of problems particularly for consistency: Do we always measure what we mean to measure? How comparable are different measures of mate choice to one another? For example, measuring preference as positive if copulation occurs is a motor act – a behavior – involving interactions with potential mates, which may be quite different from a focal individual's cognitive assessments of fitness that would be conferred by the mating.

### Is mate assessment (“choosiness”) costly?

A common definition of “choosiness” is the effort an individual is prepared to invest in mate assessment (Jennions and Petrie 1997), which assumes that assessment is often costly. The SPT explicitly assumes that any “effort” associated with cognitive assessment occurs before individuals enter receptivity the first time, or that assessment of encountered potential mates is without time costs, happening immediately on encounter with potential mates. This explicit assumption is embedded in the SPT, because there is no time‐eating state prior to encountering a potential mate during which assessment of alternative potential mates occurs. Gowaty and Hubbell (2009) informally assumed that individuals could make assessments of potential mates using information and criteria about themselves and others gained during prereproductive life stages, well before the onset of first‐time receptivity to mating. Their simplifying assumption kept the SPT simple. Nonetheless, modifying the SPT to include a time‐eating state of assessment that individuals could enter after encounter and before mating would under most conditions lower expected mean lifetime mating success and, depending on variation in the other parameters, such as the *w*‐*distribution*, increase the number of potential mates an individual would rank as acceptable, *that is,* it would move the switch point toward potential mates conferring lower fitness. In other words, the current structure of SPT suggests that after entering receptivity for the first time, investing time in assessments would likely be selected against. Future, modified, more complex versions of the SPT could include a assessment state after encountering a potential mate but before accepting or rejecting a potential mate. We could compete the new results with the results from our current versions to evaluate our intuition that selection would act against a time‐eating assessment state.

### Recommendations for the future

What is the extent of within‐individual variation in reproductive decision‐making, *that is*, in individuals of both sexes? Is it trivial in one sex, occasional or common in both sexes? Needed are study designs that include individuals independent of their expected parental investment patterns or gamete size (Naud et al. 2008). How often do individuals in “the indiscriminate sex” switch from indiscriminate to choosy? We recommend that investigators adopt (1) a symmetrical approach to simultaneously evaluate in both sexes within‐individual flexible reproductive decision‐making behavior, and (2) a more operational approach that differentiates cognitive processes from motor acts when studying variation in “choosy” and “indiscriminate” behavior (Box 1). In addition and most important, (3) we need information on whether flexible mate choice affects individual fitness (McLain 1991) is observed individual flexibility adaptive or not (McLain 2005)? Finally, (4) more investigations are needed in many more species to find out how and when males reject potential mates (Bateman and Fleming 2006).

## Attributions

P. A. Gowaty conceptualized the review. M. Ah‐King searched for papers, categorized them by topic, noting methods, and managed the bibliography. M. Ah‐King and P. A. Gowaty categorized the studies according to the parameters of the switch point theorem, M. Ah‐King summarized the studies, and P. A. Gowaty wrote the paper. M. Ah‐King and P.A. Gowaty contributed equally to the paper: M. Ah‐King is first author. P. A. Gowaty is corresponding author.

## Conflict of Interest

None declared.
